# Circulating FGF18 is decreased in pleural mesothelioma but not correlated with disease prognosis

**DOI:** 10.1111/1759-7714.15004

**Published:** 2023-06-21

**Authors:** Berta Mosleh, Karin Schelch, Thomas Mohr, Thomas Klikovits, Christina Wagner, Lukas Ratzinger, Yawen Dong, Katharina Sinn, Alexander Ries, Walter Berger, Bettina Grasl‐Kraupp, Konrad Hoetzenecker, Viktoria Laszlo, Balazs Dome, Balazs Hegedus, Marko Jakopovic, Mir Alireza Hoda, Michael Grusch

**Affiliations:** ^1^ Department of Thoracic Surgery Medical University of Vienna Vienna Austria; ^2^ Center for Cancer Research Medical University of Vienna Vienna Austria; ^3^ National Koranyi Institute of Pulmonology Budapest Hungary; ^4^ Department of Thoracic Surgery National Institute of Oncology‐Semmelweis University Budapest Hungary; ^5^ Department for Respiratory Diseases Jordanovac University of Zagreb School of Medicine, University Hospital Centre Zagreb Zagreb Croatia

**Keywords:** biomarker, FGF18, fibroblast growth factor 18, pleural mesothelioma

## Abstract

**Background:**

Pleural mesothelioma (PM) is a relatively rare malignancy with limited treatment options and dismal prognosis. We have previously found elevated FGF18 expression in PM tissue specimens compared with normal mesothelium. The objective of the current study was to further explore the role of FGF18 in PM and evaluate its suitability as a circulating biomarker.

**Methods:**

FGF18 mRNA expression was analyzed by real‐time PCR in cell lines and in silico in datasets from the Cancer Genome Atlas (TCGA). Cell lines overexpressing FGF18 were generated by retroviral transduction and cell behavior was investigated by clonogenic growth and transwell assays. Plasma was collected from 40 PM patients, six patients with pleural fibrosis, and 40 healthy controls. Circulating FGF18 was measured by ELISA and correlated to clinicopathological parameters.

**Results:**

FGF18 showed high mRNA expression in PM and PM‐derived cell lines. PM patients with high FGF18 mRNA expression showed a trend toward longer overall survival (OS) in the TCGA dataset. In PM cells with low endogenous FGF18 expression, forced overexpression of FGF18 resulted in reduced growth but increased migration. Surprisingly, despite the high FGF18 mRNA levels observed in PM, circulating FGF18 protein was significantly lower in PM patients and patients with pleural fibrosis than in healthy controls. No significant association of circulating FGF18 with OS or other disease parameters of PM patients was observed.

**Conclusions:**

FGF18 is not a prognostic biomarker in PM. Its role in PM tumor biology and the clinical significance of decreased plasma FGF18 in PM patients warrant further investigation.

## Graphical abstract

We analyzed circulating FGF18 in the plasma of 40 patients with pleural mesothelioma, six patients with pleural fibrosis and 40 healthy controls. FGF18 was significantly decreased in the plasma of patients with pleural mesothelioma and pleural fibrosis compared with healthy controls. Within the pleural mesothelioma cohort, FGF18 was not significantly correlated with overall survival or other disease parameters.

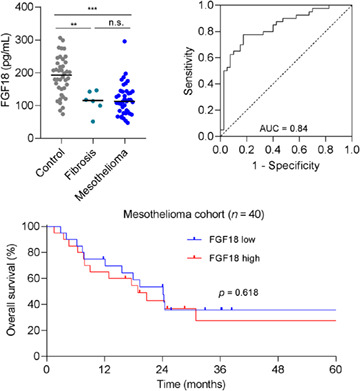

## INTRODUCTION

Pleural mesothelioma (PM) is a relatively rare malignancy with poor prognosis, limited therapeutic options, and a lack of reliable biomarkers to aid in diagnosis and patient stratification.^1^ Despite the demonstrated usefulness of mesothelin and calretinin as blood‐based biomarkers,^2^, ^3^ both the diagnosis and establishment of prognosis of PM are often challenging tasks. Accordingly, novel biomarkers are important to facilitate an earlier and more accurate diagnosis, as well as to provide prognostic information for clinicians.

FGF18 is a secreted glycoprotein and belongs to the fibroblast growth factor (FGF)/fibroblast growth factor receptor (FGFR) family that consists of 18 ligands and 4 transmembrane receptor tyrosine kinases.^4^ Various FGFR family members have been shown to play important roles in an increasing number of malignant diseases.^5^ Our group^6^, ^7^, ^8^ and others^9^, ^10^, ^11^, ^12^ have shown the importance of the FGF/FGFR signaling axis in PM in recent years. In particular, FGF2 has been reported to be overexpressed in PM cell lines and tissues and to enhance cell proliferation and epithelial to mesenchymal transition (EMT).^6^, ^7^, ^11^ Moreover, high FGF2 levels in the blood and in pleural effusions have been correlated with tumor aggressiveness and worse prognosis.^9^, ^12^ FGF18, like FGF2, is overexpressed in PM tissue specimens compared with normal pleura and has shown higher gene expression levels in PM cell lines than in normal mesothelial cells.^6^ However, in contrast to FGF2, which binds all FGFR isoforms, FGF18 has shown preferential binding to FGFR3.^13^ FGFR3 has shown a more restricted expression pattern in PM patients compared with FGFR1 and FGFR2 in a previous immunohistochemistry study and was significantly associated with poorer overall survival (OS).^8^

Physiologically, FGF18 plays a crucial role in the development of skeleton, cartilage, lung, and brain.^14^, ^15^, ^16^ With regard to malignant diseases, FGF18 has been shown to be overexpressed in several cancer types including hepatocellular, ovarian, and colorectal cancers.^17^, ^18^, ^19^, ^20^ Moreover, it has been shown to contribute to enhanced cell proliferation, migration, invasion, neoangiogenesis, and drug tolerance in a number of normal and malignant cell types.^19^, ^21^, ^22^, ^23^ In ovarian cancer, FGF18 was identified as a blood‐based biomarker by secretome analysis and enhanced FGF18 levels were confirmed by ELISA in the blood from ovarian cancer patients compared with a control group.^24^

In the current study, we further explored the role of FGF18 in pleural mesothelioma and evaluated its suitability as a blood‐based biomarker.

## METHODS

### Cell lines

Cells were cultured in growth medium with 10% fetal bovine serum (FBS) at 37°C in a humidified atmosphere with 5% CO_2_ and regularly checked for mycoplasma contamination. Cell line names, cancer type and source for each cell line are listed in Table S1. Cell line authentication was done by array comparative genomic hybridization and short tandem repeat (STR) analysis as described.^25^

### Determination of FGF18 gene expression by quantitative real‐time PCR (qRT‐PCR)

Cells were grown in flasks to about 80% confluence. Total RNA was extracted with the innuPREP RNA mini kit (Analytik Jena) according to the manufacturer's instructions. RNA was reverse transcribed with reverse transcriptase (M‐MLV, Thermo Fisher Scientific). The resulting cDNAs were used as templates for qRT‐PCR analysis with Taqman assays for FGF18 (Hs00826077m1) and GAPDH (Hs99999905m1) both from Thermo Fisher Scientific. Relative gene expression levels were calculated as 2^−dCt^ × 10^4^ of FGF18 normalized to the housekeeping gene GAPDH as previously published.^6^

### Extraction of FGF18 gene expression and survival data from the TCGA mesothelioma dataset

RNASeq expression data for FGF18 as well as clinical data were downloaded into R using the TCGAbiolinks package [https://doi.org/10.1371/journal.pcbi.1006701].

### Generation of FGF18 overexpressing cell lines

A retroviral expression construct for FGF18 was generated by amplifying the full open reading frame of FGF18 with a proofreading polymerase (Q5, New England Biolabs) and primers FGF18‐for: 5′‐TTTTTAATTAACGATGTATTCAGCGCCCTC‐3′ and FGF18‐rev: 5′‐TTTTTAATTAACCTAGGCAGGGTGTGTG‐3′ from cDNA of the PM cell line M38K and ligating the amplicon into the retroviral expression vector pQCXIP (Takara Bio). After sequence verification, viral particles were generated by transient co‐transfection of the FGF18 expression construct or the empty pQCXIP vector (as vector control) with the two helper plasmids VSV‐G and pgag‐pol‐gpt into HEK293 cells. Supernatants containing viral particles were used for target cell transduction and cells with stable integration of the FGF18 construct were selected with puromycin (0.8 μg/mL) as published.^26^ Overexpression of FGF18 was confirmed by qRT‐PCR as outlined above.

### Clonogenic growth assays

Cells were seeded at low density (10^3^ per well) into 24‐well plates and allowed to grow for up to 14 days until cell clones had formed. Then cells were washed with PBS, fixed with methanol‐glacial acetic acid (3:1) for 20 min and again washed with PBS. Afterward cells were stained with crystal violet (10% in ethanol, 1:1000 in PBS) for 1–3 h. To remove the excessive crystal violet, the plates were washed and air‐dried overnight. Images of the stained colonies were taken with a Nikon D90 camera, and afterward the cells were destained with 2% SDS for about 3 h. The solution was transferred into microtiter 96‐well plates and the absorption at 562 nm was photometrically measured with a SynergyHT plate reader.

### Migration assays

For analyzing cell migration, transwell assays using BD Falcon 8 μm pore size cell culture inserts in a 24‐well format were performed. Cells (10^4^ per well) were seeded into transwell chambers and allowed to transmigrate for 24 h. Cells that had transmigrated to the lower surface of the chamber were fixed with cold methanol for 20 min, whereas cells that remained on the upper surface were removed using a cotton stick. Afterward, cells were washed, stained with crystal violet, washed again, destained with 2% SDS and absorbance of the solution was measured at 562 nm as outlined above for the clonogenic growth assay.

### Patients

Plasma samples were collected from 40 patients with histologically confirmed PM at the time of diagnosis and/or before surgical resection. None of the patients had received talc pleurodesis before blood collection. Twenty‐nine samples were obtained at the Department of Thoracic Surgery, Medical University of Vienna. Eleven samples were collected at the University of Zagreb, School of Medicine, Department for Respiratory Diseases Jordanovac, University Hospital Center Zagreb, Croatia. Samples from 40 healthy individuals and six patients with benign pleural diseases (2 with asbestos‐induced diffuse pleural fibrosis, 3 with inflammation‐induced pleural fibrosis of unknown origin, and 1 with hyaluronan‐induced pleural fibrosis) served as controls. In all analyzed patients, PM diagnosis was histologically proven during clinical routine work‐up. The latest version of the TNM IMIG/IASLC staging system^27^ was used for clinical and pathological tumor staging. Clinical data and plasma samples were collected prospectively for all cases according to the corresponding local ethic committees of each center.

### Determination of circulating levels of FGF18

Circulating FGF18 was measured in plasma samples with the FGF18 ELISA kit from USCN (USC‐SEC907HU). Sample preparation, generation of standard curves, and measurement of samples in duplicates were done following the manufacturer's instructions.

### Statistical analysis

Categorical data are displayed as counts and percentages and metric data are given as median and interquartile range (IQR), or, in case of survival, as median and corresponding 95% confidence interval (CI) if not otherwise indicated. In the plasma sample test cohort as well as in the TCGA dataset, patients were divided into high and low FGF18 level groups by the median value (112.3 pg/mL) of protein and relative gene expression (320.9), respectively, as in previous studies.^28^, ^29^ To compare groups, Mann–Whitney U tests, Kruskal–Wallis or Chi‐square‐tests were performed as appropriate. The correlation of metric data was analyzed by Pearson's correlation coefficient. Overall survival was defined as time between diagnosis and death or last follow‐up date. Survival was estimated by the Kaplan–Meier method and log rank test. Breslow test was used to compare the group differences as appropriate. Univariate and multivariate Cox regression models were used to evaluate the effect of other potential influencing factors, such as age, sex, histology, stage, and treatment. For experiments involving cell lines, data were obtained from *n* ≥ 3 replicates and unpaired *t*‐tests were used for comparisons of two groups. Differences were considered statistically significant for *p* values <0.05. Statistical analyses were performed using the SPSS 28.0 software system (SPSS Inc.) and plots were generated with GraphPad Prism 8.

## RESULTS

### Comparison of FGF18 gene expression in pleural mesothelioma and other cancer types and correlation with survival of mesothelioma patients

**FIGURE 1:**
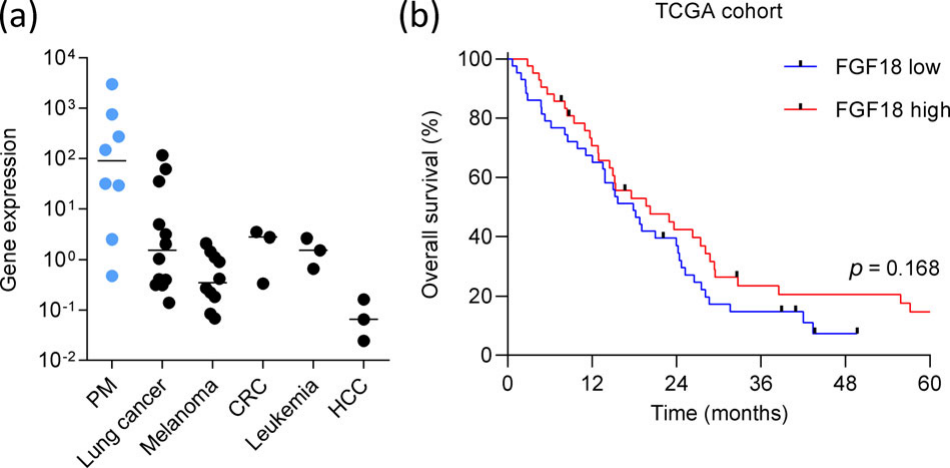
FGF18 gene expression levels are high in pleural mesothelioma (PM) cell lines and tend to be higher in mesothelioma patients with longer overall survival. (a) FGF18 gene expression was analyzed by qRT‐PCR and normalized to the housekeeping gene GAPDH in cell lines from malignant PM, lung cancer, melanoma, colorectal cancer (CRC), leukemia and hepatocellular carcinoma (HCC). Each dot represents one cell line; medians for each cancer type are indicated by horizontal lines. (b) Data were extracted from the TCGA dataset (*n* = 85). Kaplan–Meier survival analysis was performed for patients with high and low FGF18 gene expression with the median FGF18 expression level used as a cutoff.

Our previous work showing higher FGF18 expression in PM cell lines compared with mesothelial cells^6^ prompted us to compare FGF18 gene expression in PM cell lines with cell lines from other malignancies including lung cancer, colon cancer, and melanoma. Indeed, pleural mesothelioma cells showed on average the highest gene expression levels of the whole cell line panel (Figure 1a, Table S1). These data are in line with the gene expression data from the TCGA consortium (https://www.cancer.gov/about-nci/organization/ccg/research/structural-genomics/tcga), where pleural mesothelioma tissue showed the second highest FGF18 expression after ovarian cancer across 32 cancer types when analyzed on the UALCAN portal (Figure S1).^30^ Among the eight PM cell lines, no difference between those derived from epithelioid PM (*n* = 5) and those from biphasic PM (*n* = 3) and no difference between BAP1^+^ (*n* = 6) and BAP1^−^ (*n* = 2) cell lines were apparent (Figure S2).

The high FGF18 mRNA expression in PM cell lines as compared with cell lines from other cancer types suggested a potentially important role of FGF18 in PM, and therefore we next retrieved FGF18 gene expression data from the TGCA dataset of PM patients (*n* = 85). A comparison of FGF18 mRNA expression with patient survival revealed a trend towards longer OS in patients with high FGF18 (Figure 1b) which was, however, not statistically significant (median survival of 17.867 vs. 20.267 months in the FGF low vs. FGF18 high group, HR 1.397, 95% CI: 0.867–2.250, *p* = 0.168).

### Impact of FGF18 on pleural mesothelioma cell growth and migration

**FIGURE 2:**
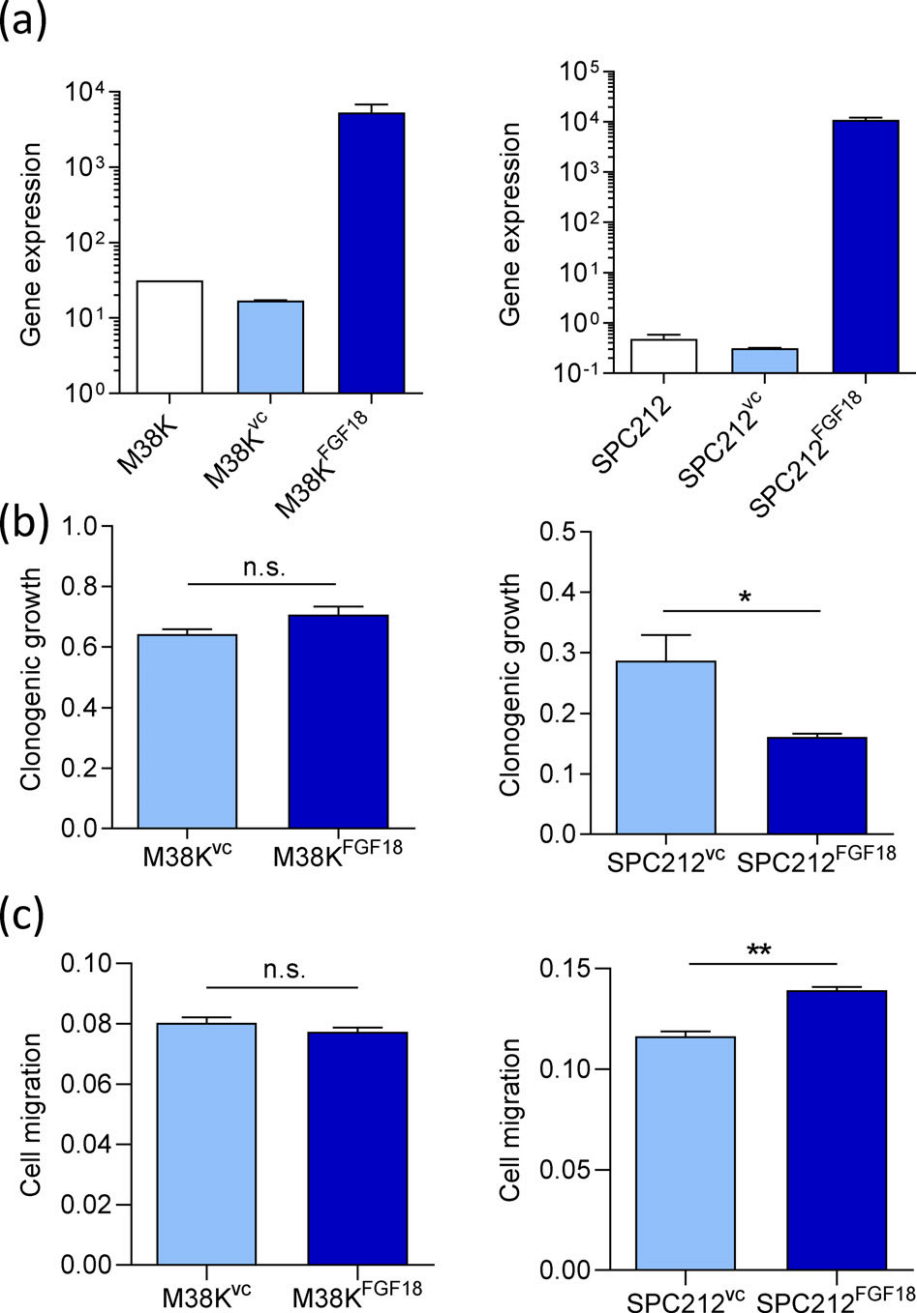
Overexpression of FGF18 results in decreased growth and enhanced migration in SPC212 but not in M38K cells. (a) FGF18 gene expression was determined in parental M38K and SPC212, corresponding vector controls (M38K^VC^, SPC212^VC^) and FGF18 overexpressing derivatives (M38K^FGF18^, SPC212^FGF18^) by qRT‐PCR. The housekeeping gene GAPDH was used for normalization. (b) Cells were seeded at low density into six‐well plates and colony formation was monitored for up to 14 days. Clonogenic growth was determined photometrically. Bars represent mean absorbance ± SEM of *n* ≥ 3 repeats. (c) Cells were seeded into transwell chambers and transmigration to the lower side of the membrane was assessed photometrically after 48 h. Bars represent mean absorbance ± SEM of *n* ≥ 3 repeats. *p < 0.05; **p < 0.01; ns, not significant; M38K^FGF18^/SPC21^FGF18^ versus the respective vector controls; unpaired *t*‐test.

Since the gene expression data suggested that FGF18 is overexpressed in PM but could be connected to longer OS, we next explored potential effects of FGF18 overexpression on PM cells. For that purpose, we selected M38K and SPC212, 2 PM cell lines with low to moderate endogenous FGF18 expression (Table S1), to generate sublines stably overexpressing FGF18 (M38K^FGF18^, SPC212^FGF18^) and the respective empty vector controls (M38K^VC^, SPC212^VC^). Parental M38K had a higher endogenous FGF18 expression than SPC212 but nevertheless strong overexpression of ectopically expressed FGF18 could be achieved in both cell lines compared with the parental cell lines as well as the respective vector controls (Figure 2a).

First, we assessed the impact of FGF18 on cell growth. While M38K^FGF18^ showed no difference to the vector control, clonogenic growth was significantly reduced in SPC212^FGF18^ compared with SPC212^VC^ (Figure 2b). We also investigated cell migration, but again found no change in response to FGF18 overexpression in the M38K cell line. SPC212^FGF18^, in contrast, showed a significant increase in cell migration (Figure 2c). Together, these findings suggest that FGF18 may influence the behavior of a subset of PM cells.

### Circulating FGF18 levels in healthy controls and patients with PM or pleural fibrosis

Since FGF18 showed some impact on PM cell behavior and, moreover, patients with high FGF18 mRNA expression in the TCGA dataset tended to have a better OS, we next analyzed circulating FGF18 protein in the plasma of PM patients in order to assess its potential suitability as a biomarker. For that purpose, we performed ELISA assays of plasma samples of 40 PM patients (median age 60.0 years, IQR: 52–69, 70% male) from two different institutions along with 40 healthy controls (median age 67.5 years, IQR: 62–72, 80% male) and 6 patients with benign pleural disease (median age 72, IQR: 61–76, 67% male).

**FIGURE 3:**
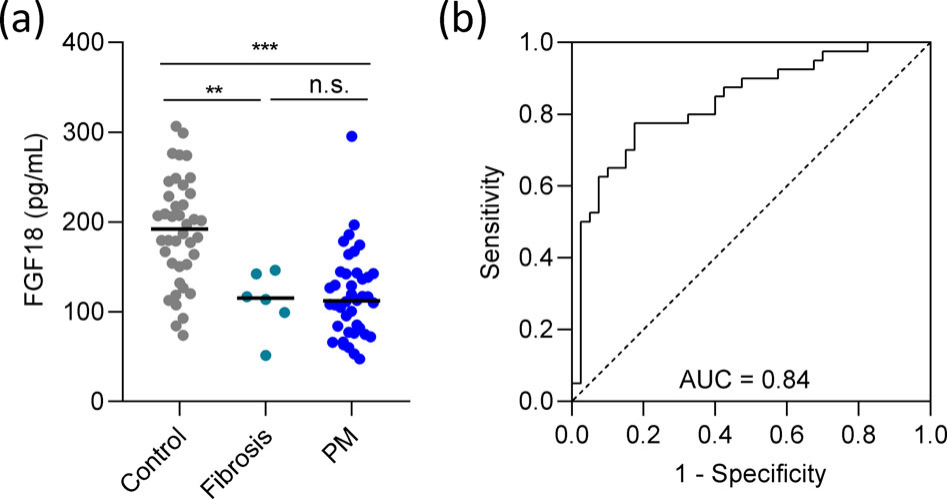
FGF18 is significantly reduced in patients with pleural mesothelioma (PM) or pleural fibrosis compared with healthy controls. (a) FGF18 levels were determined by ELISA in patient plasma and values were compared between patients with malignant PM, patients with pleural fibrosis and healthy individuals (control). Values are shown as scatter dot plots, medians are shown as horizontal lines. **p < 0.01; ***p < 0.001; ns, not significant; Kruskal–Wallis test. (b) Receiver operating characteristic (ROC) curve analysis showing the ability of FGF18 to discriminate between healthy individuals and patients with PM (AUC 0.84, 95% CI: 0.75–0.93, *p* < 0.0001).

First, the FGF18 levels were compared between patients with PM, patients with benign fibrosis and healthy individuals (Figure 3a). Surprisingly, the median FGF18 levels in PM patients were significantly lower (*n* = 40; 112.3 pg/mL, IQR: 79.7–142.5) than in healthy controls (*n* = 40; 192.4 pg/mL, IQR: 151.8–230.4; *p* < 0.001). Moreover, the median FGF18 levels were significantly lower in patients with benign fibrosis (*n* = 6, 115.5 pg/mL, IQR: 99.4–142.4) than in healthy controls (*p* = 0.004). No significant difference in FGF18 could be observed between patients with PM and patients with fibrosis (*p* = 0.974). Receiver operating characteristic (ROC) curve analysis was performed to assess the ability of FGF18 to discriminate between healthy individuals and patients with PM and showed an area under the curve of 0.837 (Figure 3b). When the six patients with fibrosis were included, the AUCs for discriminating between healthy controls and all patients (fibrosis plus PM) and between presence (PM patients) or absence (healthy controls and fibrosis patients) of malignant disease were 0.84 and 0.79, respectively (Figure S3).

### Comparison of circulating FGF18 levels with histological subtype, stage of disease and asbestos exposure

**FIGURE 4:**
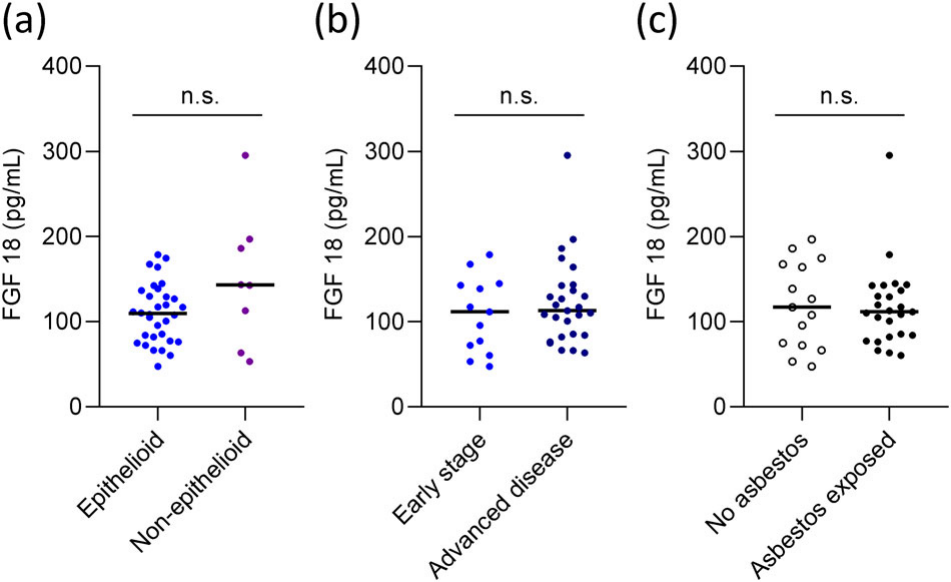
FGF18 is not significantly associated with histology, stage of disease or asbestos exposure. (a) FGF18 levels in the plasma were compared between patients with epithelioid and nonepithelioid pleural mesothelioma (PM). (b) FGF18 levels in the plasma were compared between patients with early‐stage disease (IMIG stage I and II) and patients diagnosed in an advanced stage (IMIG stage III and IV). (c) FGF18 levels in the plasma were compared between patients with and without asbestos exposure. Values are shown as scatter dot plots, medians are shown as horizontal lines. ns, not significant; Mann–Whitney U test.

Within the PM group, 26/40 (65%) patients were ≥65 years old and 28/40 (70%) were male patients. Epithelioid histology made up 80% (*n* = 32/40) of all cases. Thirteen of 40 patients presented with early‐stage disease (IMIG stage I and II), while 27/40 patients were diagnosed in an advanced stage (IMIG stage III and IV). Treatment strategies included radical surgery as part of multimodality treatment protocols (45%, *n* = 18), chemo‐ and/or radiotherapy (50%, *n* = 20) and best supportive care (5%, *n* = 2). Based on the median FGF18 value, the study cohort was divided into patients with high and low FGF18 levels. Detailed baseline characteristics of both groups are displayed as Table 1. Between low and high FGF18 groups, no significant differences in age (*p* = 0.234), sex (*p* = 0.490), histology (*p* = 0.114), stage (*p* = 0.567), and treatment (*p* = 0.329) were observed. However, there was a recognizable, not significant tendency toward higher median plasma FGF18 levels in the nonepithelioid group (*n* = 8/40; 143.0 pg/mL; IQR: 88.2–191.5; the nonepithelioid group consisted of 5 patients with biphasic PM, 2 patients with sarcomatoid PM, and 1 patient with desmoplastic PM [Figure S4]) when compared with the group with epithelioid histology (*n* = 32/40, 109.4 pg/mL; IQR: 79.7–133.2; *p* = 0.146) (Figure 4a). There was no difference in plasma FGF18 levels between patients with early‐stage disease and patients with advanced disease (Figure 4b) or between patients with (*n* = 25) and without (*n* = 15) anamnestically established asbestos exposure (Figure 4c).

**TABLE 1:** Clinicopathological characteristics of the PM patient cohort grouped by circulating FGF18.

Demographics	Study cohort (*n* = 40)	Low FGF18 (*n* = 20)	High FGF18 (*n* = 20)	*p*‐value
Age, years, median, IQR	60 (52–69)	59 (48–64)	64 (53–71)	0.234
Sex				0.490
Male	28 (70%)	15 (75%)	13 (65%)	
Female	12 (30%)	5 (25%)	7 (35%)	
Histology				0.114
Epithelioid	32 (80%)	18 (90%)	14 (70%)	
Nonepithelioid	8 (20%)	2 (10%)	6 (30%)	
Stage				0.567
I	2 (5%)	1 (5%)	1 (5%)	
II	11(27.5%)	6 (30%)	5 (25%)	
III	15 (37.5%)	9 (45%)	6 (30%)	
IV	12 (30%)	4 (20%)	8 (40%)	
Treatment				0.329
Surgery‐based MMT	18 (45%)	10 (50%)	8 (40%)	
Chemo‐ and/or radiotherapy	20 (50%)	10 (50%)	10 (50%)	
Best supportive care	2 (5%)	0 (0%)	2 (10%)	
Site				0.525
Left	18 (45%)	10 (50%)	8 (40%)	
Right	22 (55%)	10 (50%)	12 (60%)	

Abbreviations: IQR, interquartile range; MMT, multimodal treatment; PM, pleural mesothelioma.

### Correlation of circulating FGF18 levels with disease prognosis in PM

**FIGURE 5:**
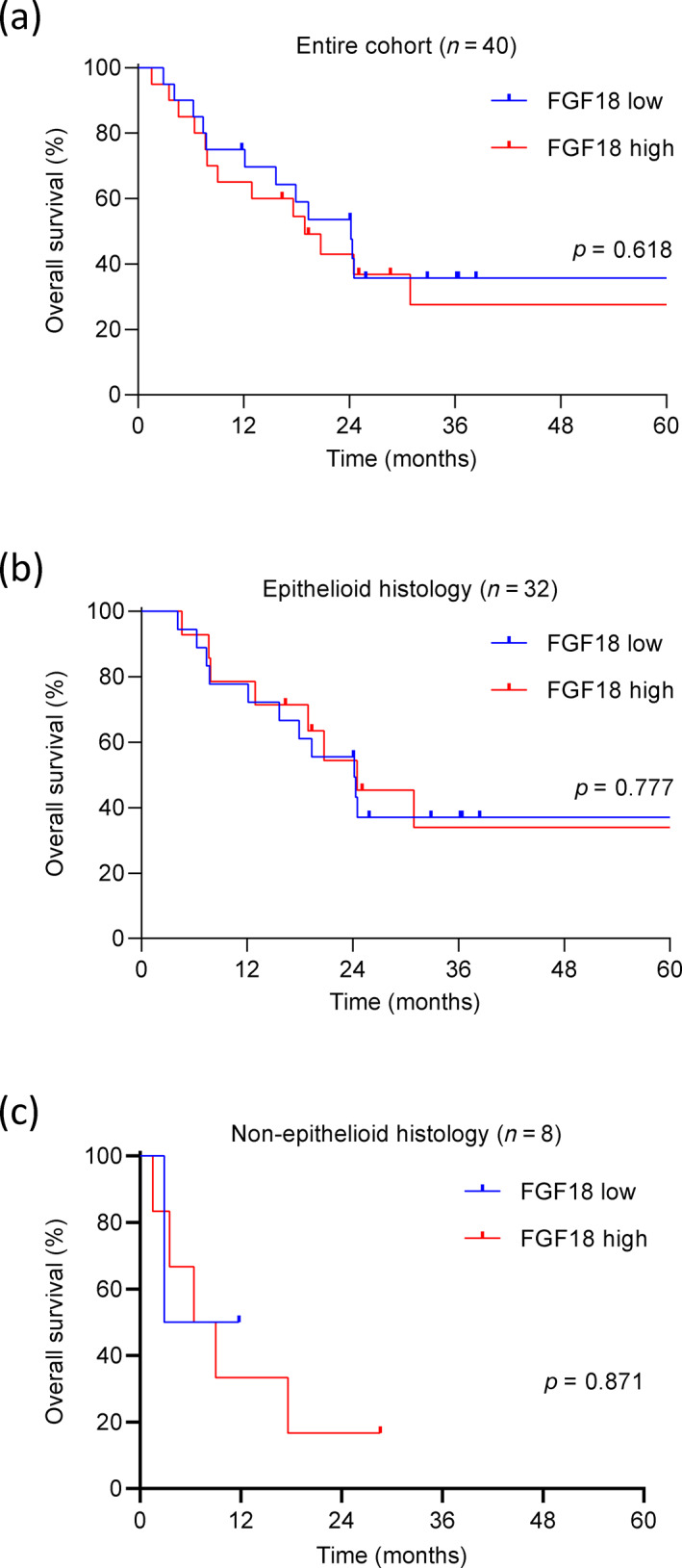
Circulating FGF18 is not associated with overall survival of pleural mesothelioma (PM) patients. (a) Kaplan–Meier survival analysis was performed for all patients of the cohort (*n* = 40) dichotomized by the median level of circulating FGF18 (112.3 pg/mL). (b) Kaplan–Meier survival analysis was performed for patients with epithelioid PM (*n* = 32). (c) Kaplan–Meier survival analysis was performed for patients with nonepithelioid PM (*n* = 8).

Finally, we tested whether levels of circulating FGF18 correlate with patient prognosis. Median OS for the entire cohort was 20.733 months (HR 3.612, 95% CI: 13.655–27.812). PM patients with low FGF18 levels had a longer, however not significantly longer overall survival when compared with those with high FGF18 levels (median survival 24.167 vs. 18.900 months, HR 0.821, 95% CI: 0. 378–1.783, *p* = 0.618) (Figure 5). We performed univariate and multivariate survival analyses including age, sex, histologic subtype, FGF18 levels, tumor site, tumor stage, and type of treatment (Table 2). Epithelioid histology held a prognostic value in univariate analysis (epithelioid vs. nonepithelioid; HR 2.905, 95% CI: 1.131–7.461, *p* = 0.027). No independent predictors, however, were detected by multivariate analysis. FGF18 was not found to be an independent prognostic factor for OS (low vs. high; HR 0.820, 95% CI: 0.330–2.039, *p* = 0.632).

**TABLE 2:** Univariate and multivariate survival analyses of the PM patient cohort.

Variables			Univariate			Multivariate
	*n* = 40	OS (CI)	*p*‐value	HR	95% CI	*p*‐value
**Age**			0.602			0.678
<65	26	24.30 (17.05–31.55)		0.66	0.49–0.89	
>65	14	15.63 (5.48–25.79)		1.51	1.12–2.05	
**Sex**			0.453			0.403
Male	28	24.30 (18.98–29.62)		1.35	0.95–1.92	
Female	12	17.60 (13.76–21.45)		0.74	0.52–1.05	
**Histology**			0.027			0.086
Epithelioid	32	24.3 (18.29–30.32)		2.91	1.13–7.46	
Nonepithelioid	8	6.4 (0.00–14.07)		0.34	0.13–0.88	
**FGF18 levels**			0.618			0.632
High	20	18.90 (8.67–29.13)		0.83	0.38–1.78	
Low	20	24.17 (15.75–32.58)		1.22	0.56—2.65	
**Site**			0.452			0.904
Right	22	19.33 (12.65–26.02)		1.19	0.88–1.60	
Left	18	24.30 (12.69–35.94)		0.84	0.62–1.14	
**Stage**			0.132			0.389
Early	13	24.47		2.02	0.81–5.05	
Late	27	18.90 (12.91–24.89)		0.50	0.20–1.24	
**Treatment**			0.227			0.599
Surgery‐based MMT	18	24.30 (18.27–30.33)		1		
CTX and/or RTX	20	12.90 (0.00–27.96)		2.19	1.56–3.06	
BSC	2	1.53 (n/a)		4.00	2.48–6.46	

Abbreviations: BSC, best supportive care; CI, confidence interval; CTX, chemotherapy; HR, hazard ratio; MMT, multimodal treatment; PM, pleural mesothelioma; OS, overall survival; RTX, radiotherapy.

## DISCUSSION

Growth factors and their receptors can have multiple functions in the development and progression of cancer. They are frequently overexpressed compared with normal tissues and, in addition to stimulating tumor cell proliferation, they have been shown to influence migration and invasion, neoangiogenesis and immune cell functions.^31^ This makes them potential candidates both as biomarkers and as therapeutic targets, especially since growth factor receptors are often kinases that can be blocked with specific kinase inhibitory drugs. In mesothelioma, several growth factors have been demonstrated to contribute to cancer progression, influence prognosis or predict the response to specific therapies. For instance, our group has previously demonstrated that activin A, a member of the TGF‐β family, drives PM growth^32^ and high circulating levels were associated with larger tumor volume and conferred a significantly worse OS in patients with epithelioid PM.^28^ TGF‐β itself was associated with shorter OS when detected in pleural effusions but not when detected in blood.^33^ Among the growth factors of the FGF family, high FGF2 was found to have negative prognostic impact in PM both in blood and pleural effusions.^9^, ^12^ In one previous study in PM, FGF9 and FGF18 gene expression were connected to loss of BAP1, a key tumor suppressor in PM, which in turn was suggested to indicate an increased sensitivity to the FGFR inhibitor AZD4547.^34^ Of the two cell lines with BAP1 loss in our cell line panel, one showed high and one low FGF18 expression. For our patient cohort, no BAP1 status was available. Altered FGF18 expression has been reported in a number of different cancer types and its presence has been connected to both pro‐ and antitumorigenic activities. Our group has previously described overexpression of FGF18 in melanoma cells compared with primary melanocytes^35^ and demonstrated a role in tumor progression in colon cancer via autocrine stimulation of tumor cells and paracrine stimulation of colon‐associated fibroblasts and endothelial cells.^19^ Enhancement of tumor progression by FGF18 has also been shown in ovarian cancer and hepatocellular carcinoma.^20^, ^21^ In previous breast cancer studies, FGF18 gene expression was included in a five gene prognostic signature for disease free survival^36^ and FGF18 enhanced breast cancer cell migration, invasion and EMT.^37^, ^38^ In gastroesophageal adenocarcinoma,^39^ in contrast, as well as in clear cell renal cell cancer,^40^ high FGF18 expression was found to be correlated with longer patient survival. In the latter case, FGF18 overexpression was, in addition, shown to inhibit proliferation and invasion of tumor cells in vitro and in vivo. In PM, we have previously found overexpression of FGF18 in tissue specimens and cell lines when compared with normal pleura cells.^6^ High gene expression of FGF18 was confirmed in the current study in PM cell lines compared with most cell lines from other malignancies and is in line with the TCGA gene expression comparison across multiple cancer types. Surprisingly, not only did this not result in increased circulating FGF18 levels in PM patients compared with healthy controls, but PM patients even exhibited significantly lower levels of FGF18 than healthy controls. A possible explanation for this seemingly contradictory finding could be that a high number of FGFRs on PM cells as reported by us^6^, ^8^ and others^10^, ^34^ might lead to a rapid internalization of receptor‐bound FGF18 and result in a more efficient clearing of FGF18 from the circulation.

While FGF18 was able to discriminate between healthy individuals and patients with PM, the lack of a significant difference between PM patients and patients with pleural fibrosis may limit its usefulness for diagnostic purposes. Several blood‐based diagnostic biomarkers including mesothelin, calretinin, osteopontin, fibulin‐3 and high‐mobility group box 1 (HMGB1) have been proposed for PM.^2^, ^3^, ^41^ A recent meta‐analysis of diagnostic biomarkers in PM concluded that mesothelin, despite being by far the most investigated diagnostic biomarker in PM with ROC curve analyses showing AUCs >0.8 across multiple studies in serum, plasma or pleural effusions, lacks the sensitivity to be used as standalone biomarker.^41^ Marker panels such as mesothelin, thioreduxin (TRX) and fibulin‐3 in serum or mesothelin, calretinin and megakaryocyte potentiating factor (MTF) in plasma could help to improve performance.^41^, ^42^, ^43^ While plasma FGF18 clearly does not represent a standalone biomarker for FGF18 diagnosis, its decrease in patients with pleural disease is an interesting finding and suggests further evaluation in combination with additional markers.

With respect to tumor biology, the effects of FGF18 reported in the literature are tissue‐type specific. Our results show that overexpression of FGF18 in PM cells with very low endogenous FGF18 can result in decreased clonogenicity. This aligns with data from renal cell cancer^40^ but contrasts with our previous results in colon cancer, where a strong stimulation of cell growth was found.^19^ The cell model that showed decreased growth also showed a moderate increase in cell migration, which would be in agreement with the “go or grow hypothesis,” although a previous report dismissed this hypothesis for unstimulated mesothelioma cells.^44^ Whether the net effect of these activities of FGF18 in vitro would favor or impair tumor progression in vivo remains unclear at present. Predominance of a growth limiting effect of FGF18 would suggest that its observed overexpression in PM cells could be a passenger effect rather than a driving event of tumorigenesis in PM, but might explain the trend towards longer OS in patients with high FGF18 gene expression observed in the TCGA dataset. These gene expression data prompted us to further investigate FGF18 as a prognostic biomarker in the circulation of patients. The subsequent ELISA analysis, however, revealed no significant correlation of FGF18 with OS or other clinicopathological parameters, essentially invalidating FGF18 as a blood‐based prognostic marker in PM. It must be emphasized, however, that some of our results, especially those concerning patients with fibrosis and nonepithelioid PM are based on small sample numbers, which is a limitation of the current study. The findings for FGF18 are in contrast to its putative receptor FGFR3, which correlated with shorter OS in PM when analyzed by IHC in tissue specimens.^8^ Data from gastric cancer suggest that FGF18 can also enact strong protumorigenic functions via FGFR2.^45^ In PM, FGFR2 upregulation was connected to loss of the tumor suppressor NF2,^46^ which is inactivated in around 20% of PM patients.^47^ Tissue expression of FGFR2 in PM, however, had no prognostic power.^8^

Overall, our data disprove circulating FGF18 as a prognostic biomarker in PM. The decrease of circulating FGF18 in pleural disease and the role of FGF18 in PM biology should be further evaluated.

## AUTHOR CONTRIBUTIONS

Conceptualization, B.H., MA.H., M.G.; software, T.M.; investigation, B.M., K.Sc., T.K., C.W., L.R., Y.D., K.Si., A.R.; resources, W.B., B.G.K., K.H., B.D.; data curation, B.M.; writing—original draft preparation, B.M., T.M., M.A.H., M.G.; writing—review and editing, all authors; visualization, B.M., M.G.; supervision, V.L., B.H., M.J.; funding acquisition, B.D. and M.G. All authors have read and agreed to the published version of the manuscript.

## CONFLICT OF INTEREST STATEMENT

The authors declare no conflict of interest. The funders had no role in the design of the study; in the collection, analyses, or interpretation of data; in the writing of the manuscript, or in the decision to publish the results.

## Supporting information


**Table S1.** Cell lines used in this study.
**Figure S1.** Mesothelioma shows the second highest FGF18 gene expression across 32 cancer types analyzed by the TCGA consortium. ACC, adrenocortical carcinoma; BLCA, bladder urothelial carcinoma; BRCA, breast invasive carcinoma; CESC, cervical squamous cell carcinoma; CHOL, cholangiocarcinoma; COAD, colon adenocarcinoma; DLBC, lymphoid neoplasm diffuse B cell lymphoma; ESC, esophageal carcinoma; GBM, glioblastoma multiforme; HNSC, head and neck squamous cell carcinoma; KICH, kidney chromophobe tumor; KIRC, kidney renal clear cell carcinoma; KIRP, kidney renal papillary cell carcinoma; LGG, brain lower grade glioma; OV, ovarian serous cystadenocarcinoma; MESO, mesothelioma; LIHC, liver hepatocellular carcinoma; LUAD, lung adenocarcinoma; LUSC, lung squamous cell carcinoma; PAAD, pancreatic adenocarcinoma; PRAD, prostate adenocarcinoma; PCPG, pheochromocytoma and paraganglioma; READ, rectal adenocarcinoma; SARC, sarcoma; SKCM, skin cutaneous melanoma; LAML, acyte myeloid leukemia; TGCT, testicular germ cell tumors; THCA, thyroid carcinoma; THYM, thymoma; STAD, stomach adenocarcinoma; UCEC, uterine corpus endometrial carcinoma; UCS, tterine carcinosarcoma; UVM, uveal melanoma. Image downloaded from http://ualcan.path.uab.edu/cgi-bin/Pan-cancer.pl?genenam=FGF18 on February 02, 2023.
**Figure S2.** FGF18 expression in cell lines from different PM subtypes. FGF18 gene expression levels show no apparent differences between the five cell lines from epithelioid and the three cell lines from biphasic PM (left panel) and the six BAP1^+^ and the two BAP1^−^ cell lines of the PM cell line panel (right panel).
**Figure S3.** Receiver operating characteristics (ROC) curves for different cohorts. ROC curves showing the sensitivity and specificity of circulating FGF18 to discriminate between healthy individuals and patients with either PM or pleural fibrosis (upper panel, AUC 0.84; 95% CI: 0.76–0.93; *p* < 0.0001) and between persons with malignant disease (PM) or without malignant disease (healthy controls and patients with pleural fibrosis) (lower panel, AUC 0.79, 95% CI: 0.70–0.89, *p* < 0.0001).
**Figure S4.** FGF18 levels in the plasma of patients with epithelioid, biphasic, sarcomatoid, and desmoplastic PM. Values are shown as scatter dot plots, medians are shown as horizontal lines. ns, not significant; Mann–Whitney U test. For sarcomatoid and desmoplastic PM, no statistical tests could be performed due to small sample numbers.

## Data Availability

All data are contained in the article and its supplementary files.
