# Supplementary material for: Circulating FGF18 is decreased in pleural mesothelioma but not correlated with disease prognosis
Source: Thorac Cancer. 2023 Jun 21;14(22):2177–86. doi: 10.1111/1759-7714.15004 (PMC10396789; doi:10.1111/1759-7714.15004)
Supplement: Supplementary file 1 — Table S1. Cell lines used in this study. Figure S1. Mesothelioma shows the second highest FGF18 gene expression across 32 cancer types analyzed by the TCGA consortium. ACC, adrenocortical carcinoma; BLCA, bladder urothelial carcinoma; BRCA, breast invasive carcinoma; CESC, cervical squamous cell carcinoma; CHOL, cholangiocarcinoma; COAD, colon adenocarcinoma; DLBC, lymphoid neoplasm diffuse B cell lymphoma; ESC, esophageal carcinoma; GBM, glioblastoma multiforme; HNSC, head and neck squamous cell carcinoma; KICH, kidney chromophobe tumor; KIRC, kidney renal clear cell carcinoma; KIRP, kidney renal papillary cell carcinoma; LGG, brain lower grade glioma; OV, ovarian serous cystadenocarcinoma; MESO, mesothelioma; LIHC, liver hepatocellular carcinoma; LUAD, lung adenocarcinoma; LUSC, lung squamous cell carcinoma; PAAD, pancreatic adenocarcinoma; PRAD, prostate adenocarcinoma; PCPG, pheochromocytoma and paraganglioma; READ, rectal adenocarcinoma; SARC, sarcoma; SKCM, skin cutaneous melanoma; LAML, acyte myeloid leukemia; TGCT, testicular germ cell tumors; THCA, thyroid carcinoma; THYM, thymoma; STAD, stomach adenocarcinoma; UCEC, uterine corpus endometrial carcinoma; UCS, tterine carcinosarcoma; UVM, uveal melanoma. Image downloaded from http://ualcan.path.uab.edu/cgi-bin/Pan-cancer.pl?genenam=FGF18 on February 02, 2023. Figure S2. FGF18 expression in cell lines from different PM subtypes. FGF18 gene expression levels show no apparent differences between the five cell lines from epithelioid and the three cell lines from biphasic PM (left panel) and the six BAP1+ and the two BAP1− cell lines of the PM cell line panel (right panel). Figure S3. Receiver operating characteristics (ROC) curves for different cohorts. ROC curves showing the sensitivity and specificity of circulating FGF18 to discriminate between healthy individuals and patients with either PM or pleural fibrosis (upper panel, AUC 0.84; 95% CI: 0.76–0.93; p < 0.0001) and between persons with malignant disease ( [file TCA-14-2177-s001.pdf]

# Circulating FGF18 is decreased in pleural mesothelioma but not correlated with disease prognosis

**Berta MOSLEH [1], Karin SCHELCH [1,2], Thomas MOHR [2], Thomas KLIKOVITS [1], Christina WAGNER [2], Lukas RATZINGER[2], Yawen DONG [1], Katharina SINN [1], Alexander RIES [2], Walter BERGER [2], Bettina GRASL-KRAUPP [2], Konrad HOETZENECKER [1], Viktoria LASZLO [1], Balazs DOME [1,3,4], Balazs HEGEDUS [1], Marko JAKOPOVIC [5], Mir Alireza HODA [1] and Michael GRUSCH [2,*]**

[1] Medical University of Vienna, Department of Thoracic Surgery, Vienna, Austria

[2] Medical University of Vienna, Center for Cancer Research, Vienna, Austria

[3] National Koranyi Institute of Pulmonology, Budapest, Hungary

[4] National Institute of Oncology-Semmelweis University, Department of Thoracic Surgery, Budapest, Hungary

[5] University of Zagreb School of Medicine, Department for Respiratory Diseases Jordanovac, University Hospital Centre Zagreb, Zagreb, Croatia

* Correspondence: michael.grusch@meduniwien.ac.at; +431 40160 57556, Medical University of Vienna, Center for Cancer Research, Borschkegasse 8a, A-1090 Vienna, Austria

**Running Title:** FGF18 in pleural mesothelioma

**Supplementary Material**

Supplementary Table S1

Supplementary Figures S1-S4

**Supplementary Table S1:** Cell lines used in this study

| Cell Line | Cancer Type | Source | FGF18 gene expression* |
|---|---|---|---|
| CRL-5820 | Pleural Mesothelioma[e] | ATCC | 2.5 |
| I2 | Pleural Mesothelioma[e] | University of Milano, A Catania | 3027.9 |
| M38K | Pleural Mesothelioma[b] | University of Helsinki, VL Kinnula | 31.7 |
| P31 | Pleural Mesothelioma[e] | University of Umea, K Grankvist | 29.2 |
| SPC212 | Pleural Mesothelioma[b] | University of Zurich, R Stahel | 0.5 |
| SPC111 | Pleural Mesothelioma[b] | University of Zurich, R Stahel | 754.0 |
| VMC6 | Pleural Mesothelioma[e] | Medical University of Vienna | 272.7 |
| VMC20 | Pleural Mesothelioma[e] | Medical University of Vienna | 149.5 |
| A-427 | Lung Cancer | ATCC | 35.5 |
| A549 | Lung Cancer | ATCC | 0.4 |
| Calu-3 | Lung Cancer | ATCC | 0.1 |
| SK-LU-1 | Lung Cancer | ATCC | 2.0 |
| VL1 | Lung Cancer | Medical University of Vienna | 0.3 |
| VL4 | Lung Cancer | Medical University of Vienna | 62.1 |
| VL5 | Lung Cancer | Medical University of Vienna | 116.7 |
| VL6 | Lung Cancer | Medical University of Vienna | 3.2 |
| VL7 | Lung Cancer | Medical University of Vienna | 0.4 |
| VL8 | Lung Cancer | Medical University of Vienna | 1.0 |
| VL10 | Lung Cancer | Medical University of Vienna | 5.0 |
| Caco-2 | Colon Cancer | ATCC | 2.8 |
| HCT15 | Colon Cancer | ATCC | 3.5 |
| HT29 | Colon Cancer | ATCC | 0.3 |

| | | | |
|---|---|---|---|
| HOS | Osteosarcoma | ATCC | 8.8 |
| MG-63 | Osteosarcoma | ATCC | 251.2 |
| HL-60 | Leukemia | ATCC | 1.5 |
| Jurkat | Leukemia | ATCC | 0.7 |
| K-562 | Leukemia | ATCC | 2.6 |
| AKH12 | Liver Cancer | Medical University of Vienna | 0.1 |
| AKH3p | Liver Cancer | Medical University of Vienna | 0.2 |
| HepG2 | Liver Cancer | ATCC | 0.02 |
| VM1 | Melanoma | Medical University of Vienna | 1.5 |
| VM8 | Melanoma | Medical University of Vienna | 1.1 |
| VM10 | Melanoma | Medical University of Vienna | 0.2 |
| VM23 | Melanoma | Medical University of Vienna | 0.9 |
| VM24 | Melanoma | Medical University of Vienna | 0.4 |
| VM28 | Melanoma | Medical University of Vienna | 0.1 |
| VM30 | Melanoma | Medical University of Vienna | 0.3 |
| VM31 | Melanoma | Medical University of Vienna | 2.1 |
| VM47 | Melanoma | Medical University of Vienna | 0.2 |
| VM48 | Melanoma | Medical University of Vienna | 0.1 |

* qRT-PCR values normalized to the housekeeping gene GAPDH, [e] epitheliod subtype, [b] biphasic subtype

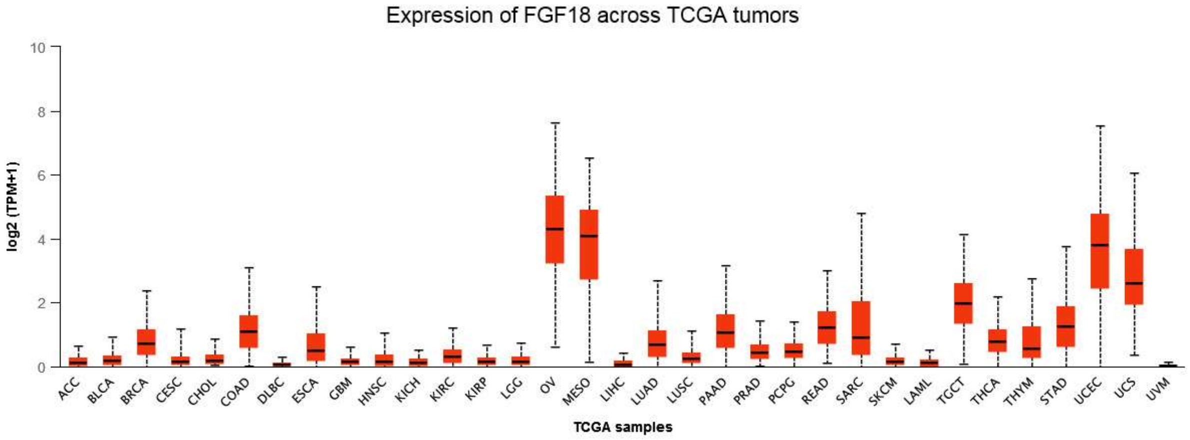


**Supplementary Figure S1:** Mesothelioma shows the second highest FGF18 gene expression across 32 cancer types analyzed by the TCGA consortium. ACC…Adrenocortical carcinoma, BLCA…Bladder urothelial carcinoma, BRCA…Breast invasive carcinoma, CESC…Cervical squamous cell carcinoma, CHOL…Cholangiocarcinoma, COAD…Colon adenocarcinoma, DLBC…Lymphoid neoplasm diffuse B-cell lymphoma, ESC…Esophageal carcinoma, GBM…Glioblastoma multiforme, HNSC…Head and neck squamous cell carcinoma, KICH…Kidney chromphobe tumor, KIRC…Kidney renal clear cell carcinoma, KIRP…Kidney renal papillary cell carcinoma, LGG…Brain lower grade glioma, OV…Ovarian serous cystadenocarcinoma, MESO…Mesothelioma, LIHC…Liver hepatocellular carcinoma, LUAD…Lung adenocarcinoma, LUSC…Lung squamous cell carcinoma, PAAD…Pancreatic adenocarcinoma, PRAD…Prostate adenocarcinoma, PCPG…Pheochromocytoma and paraganglioma, READ…Rectal adenocarcinoma, SARC…Sarcoma, SKCM…Skin cutaneous melanoma, LAML…Acyte myeloid leukemia, TGCT…Testicular germ cell tumors, THCA…Thyroid carcinoma, THYM…Thymoma, STAD…Stomach adenocarcinoma, UCEC…Uterine corpus endometrial carcinoma, UCS…Uterine carcinosarcoma, UVM…Uveal melanoma. Image downloaded from http://ualcan.path.uab.edu/cgi-bin/Pan-cancer.pl?genenam=FGF18 on 2023-02-02.

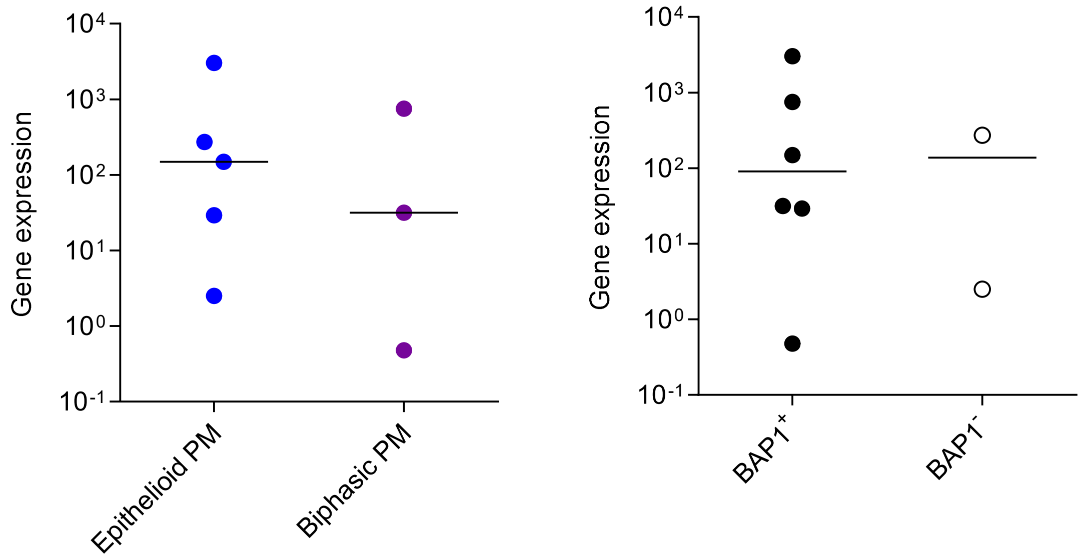


**Supplementary Figure S2:** FGF18 expression in cell lines from different PM subtypes. FGF18 gene expression levels show no apparent differences between the five cell lines from epithelioid and the three cell lines from biphasic PM (left panel) and the six $BAP1^{+}$ and the two BAP1- cell lines of the PM cell line panel (right panel).

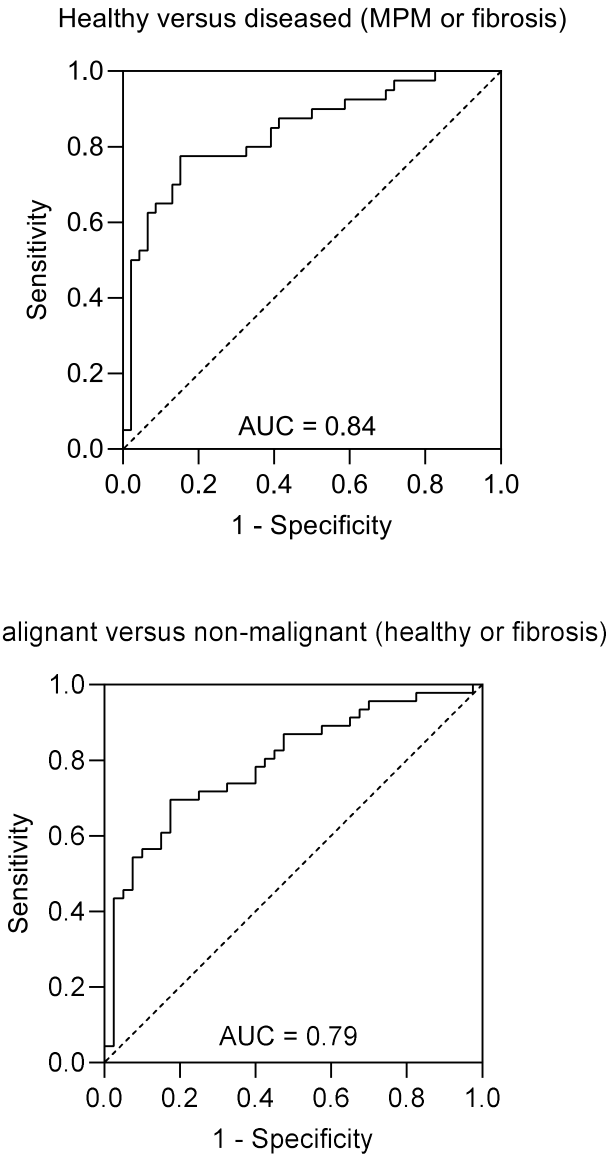


**Supplementary Figure S3:** Receiver operating characteristics (ROC) curves for different cohorts. ROC curves showing the sensitivity and specificity of circulating FGF18 to discriminate between healthy individuals and patients with either PM or pleural fibrosis (upper panel, AUC 0.84; 95% CI, 0.76-0.93; P<0.0001) and between persons with malignant disease (PM) or without malignant disease (healthy controls and patients with pleural fibrosis) (lower panel, AUC 0.79, 95% CI 0.70-0.89, P<0.0001).

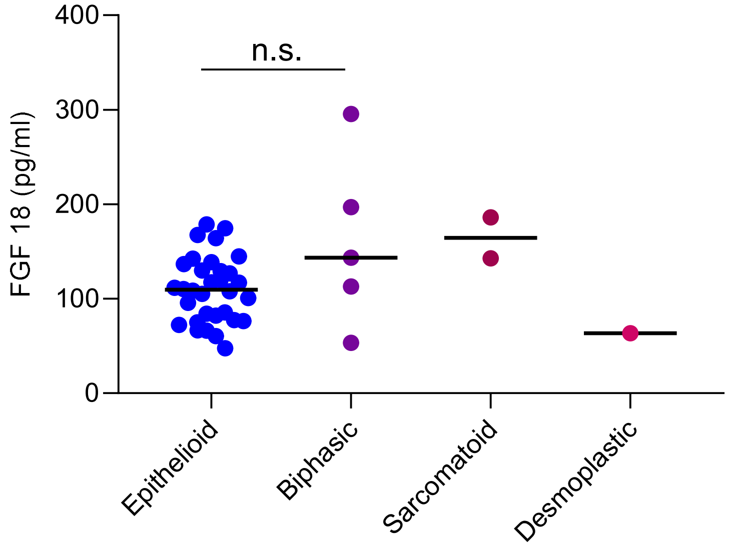


**Supplementary Figure S4:** FGF18 levels in the plasma of patients with epithelioid, biphasic, sarcomatoid and desmoplastic PM. Values are shown as scatter dot plots, medians are shown as horizontal lines. ns… not significant; Mann-Whitney U-test. For sarcomatoid and desmoplastic PM, no statistical tests could be performed due to small sample numbers.
